# Recommendations From a Health Care Provider’s Perspective for Better Implementation of Telemedicine and Telenursing: Descriptive Qualitative Approach

**DOI:** 10.2196/90610

**Published:** 2026-09-09

**Authors:** Rr Tutik Sri Hariyati, Min-Huei Hsu, Hanny Handiyani, Muhammad Hanif Amiruddin, Tuti Afriani, Dara Ayu Wardhani, Satria Dwi Pranoto, Andi Amalia Wildani, Arieny Rizafni, Citra Hafilah Shabrina

**Affiliations:** 1 Faculty of Nursing, Universitas Indonesia, Health Cluster, Building E7, Depok, West Java, 16425, Indonesia, 62 085782088101; 2 Hospital of Universitas Indonesia, Depok, West Java, Indonesia; 3 Graduate Institute of Data Science, College of Management, Taipei Medical University, Taipei, Taiwan; 4 International P.hD. Program in Biotech and Healthcare Management, College of Management, Taipei Medical University, Taipei, Taiwan

**Keywords:** human resources, 5M, method, man, money, material, and machine, telemedicine, telenursing, regulation

## Abstract

**Background:**

Digital technology-based health care services like telemedicine and telenursing are expanding quickly globally. Although the use of telemedicine and telenursing has shown promise, there are a number of obstacles to overcome.

**Objective:**

This study aimed to determine the experiences of stakeholders with using telenursing and telemedicine during and after the COVID-19 pandemic.

**Methods:**

This study combined descriptive qualitative analysis using a focus group discussion (FGD) and validation using a review of the literature. The literature search used a convenience approach with the keywords benefits, advantages, disadvantages, obstacles, and constraints of telenursing or telemedicine to find relevant journal articles. Two FGDs were conducted during the COVID-19 period, namely 2020‐2022, with the first FGD held in 2022. The second FGD was conducted in 2023‐2024 to determine the implementation of telenursing and telemedicine during the recovery period, with data collection in 2024. The FGDs were conducted via the online Zoom platform, and a total of 23 participants were selected based on purposive sampling. People involved in the development and implementation of telemedicine or telenursing were included. Data analysis was conducted in 2 stages: the first using an inductive method, which was followed by triangulation using a deductive method. The results were verified and validated based on the reviewed literature.

**Results:**

We identified three themes: (1) health care providers have experience using telemedicine/telenursing; (2) telemedicine and telenursing have many benefits; and (3) telemedicine and telenursing have 5M (method, man, money, material, and machine) factors that must be anticipated. Key statements were identified and validated, then matched with the literature review.

**Conclusions:**

The benefits and barriers of telemedicine and telenursing were identified. For telemedicine to run smoothly, preparation of 5M elements is necessary. Implications for health care services necessitate the creation of operational guidelines for the implementation of telemedicine and telenursing.

## Introduction

The role of digital health technology in all countries grew rapidly during the COVID-19 pandemic. Telemedicine and telenursing, which provide remote service facilities and can reduce physical contact that causes transmission of the COVID-19 virus, were very important during the COVID-19 pandemic [1,2]. The use of telemedicine when treating patients with COVID-19 allows consultations and administering drugs without going to health facilities, strengthening the health system. This is beneficial for the handling of COVID-19 because it can reduce the number of COVID-19 exposures [3]. Some telemedicine and telenursing technologies were used in the COVID-19 era for treatment consultations, monitoring, follow-up after hospitalization, providing counseling during isolation, and providing health education [4].

In Indonesia, telemedicine and telenursing are needed due to its archipelagic geography and numerous seas. Digital systems are essential for delivering services, as traveling to health care facilities can take more than 1 hour. Several studies provide evidence that telemedicine and telenursing are beneficial for increasing access and connecting urban and urban areas [5,6]. Telenursing can be used to direct when to take medications, to describe how to treat wounds, to help patient movement, and as a medium for health consultation for patients [5,7-9]. Facilitating the reach to more areas can save transportation and direct health care costs [10-14].

In line with the results of previous studies, telenursing can be used to monitor patients and provide coverage of services while reducing exposure to infection [10]. Several other studies also conveyed that telemedicine and telenursing can improve quality of life, reduce service costs, and be time efficient because patients do not have to go to health services; in addition, it can reduce the costs of consultation between health workers [11].

Since telenursing’s readiness frequently affects success, a 5M (man, money, method, material, and machine) preparation strategy is crucial. This 5M strategy was developed to prepare for telenursing and telemedicine. Poor community literacy competency and inadequate infrastructure limit the use of telemedicine [12,14,15]. There are many obstacles to the use of telenursing and telemedicine, such as limited patient digital literacy [14,16], and a lack of facilities can decrease the enthusiasm for telemedicine after the COVID-19 era [13,14]. The absence of full regulations, such as a credentialing process for the telemedicine service provider industry, is an obstacle in Indonesia. A previous study stated that regulation was needed for telemedicine implementation. In addition, certification and credentialing for human resources who perform telemedicine practices are not available. The issue of patient data security and confidentiality is also present in Indonesian telehealth practices [12,13].

In some areas, the use of telehealth, including telemedicine and telenursing, decreased after the COVID-19 era due to patient preferences [14,17]. This online consultation model boomed in the COVID-19 era, but has gradually decreased since. Many consumers returned to direct services because they could meet directly with care providers such as doctors and nurses. According to prior research, a more accurate disease diagnosis, more accurate and better patient examination by a physician, and more accurate and better disease treatment are the primary reasons for selecting in-person appointments [17,18]. This study aimed to determine the experiences of stakeholders with using telenursing and telemedicine during and after the COVID-19 pandemic. The study also aimed to identify the readiness of 5M aspects to support telenursing implementation. The research results were expected to provide recommendations for the ideal implementation of telenursing and telemedicine.

## Methods

### Study Design

This study used a descriptive qualitative technique to investigate the perceptions of health stakeholders for managing the advantages of telemedicine and telenursing and to determine the weaknesses of 5M factors. In this study, we excluded market aspects and focused our evaluations on how workflows (methods), people, money, machines, and materials were executed.

The study results will provide recommendations for better telemedicine and telenursing implementation by conducting a synthesis that combines a literature review and descriptive qualitative analysis of focus group discussions (FGDs). We searched for journal articles using a convenience approach with the following keywords: benefits, advantages, disadvantages, 5M obstacles, and constraints of telenursing or telemedicine. We posed 4 questions regarding the use of telenursing and telemedicine during 2 data collection periods, namely during the final period of the COVID-19 pandemic (2022‐2023) and after the recovery period (2024).

We conducted 2 FGDs. The first was held in 2022, to represent the COVID-19 period (2020‐2022). The second FGD was conducted in 2024 to determine the implementation of telenursing and telemedicine during the recovery period (2023-2024). The FGDs were conducted via the online Zoom platform. Data collection was conducted twice to examine perspectives during and after the COVID-19 pandemic. After the COVID-19 pandemic, there was a tendency for patients to prefer direct care at health facilities.

The questions asked during the FGDs were almost the same, with the aim of understanding the perspectives, benefits, and potential challenges that arise during the implementation of telehealth. These questions were as follows: (1) Describe the telemedicine or telenursing services you have utilized. (2) What are the prospects for telemedicine and telenursing implementation? (3) Could you explain the shortcomings experienced and 5M factors when using telemedicine and telenursing? and (4) What do you hope to achieve by utilizing telemedicine or telenursing?

### Participants and Approach to Their Selection

Data were gathered through semistructured FGD with policymakers, hospital organizations, medical doctors, nurses, and health care providers. Purposeful sampling was used to ensure representation across different occupations and demographic groups. Participants were invited by sending a letter of application to become a participant to hospitals, clinics, health centers and health worker associations. The organization then appointed the sections that would participate in the FGD according to the requested criteria, namely having experience in implementing digital health, including telemedicine or telenursing.

We selected 12 FGD participants in 2022 and 11 FGD participants in 2024 via purposive sampling. Ideally, the FGD participants would have been the same individuals, but this study had a weakness in that participants in the first FGD could not participate in the second FGD. Participants in 2022 and 2024 were different people because they were sent by the target organizations according to the research objectives. Some differences in the participants were due to changes in personnel and tasks of the organization during the COVID-19 period. The professions of the participants were diverse, namely nurses, midwives, doctors, lecturers, medical recorders, lawyers, and others. Additionally, involving a variety of stakeholders was expected to help identify potential and 5M barriers to telemedicine and to develop targeted recommendations to address them effectively.

### Data Collection

Response bias was possible because the data were gathered through focus group conversations, where individuals might alter their answers in reaction to what other group members think. To reduce prejudice, the facilitator gave each participant information at the outset so they may voice their opinions independently of other participants’ experiences. At the conclusion of the conversation, each member had the opportunity to share personal experiences that had not been discussed. Saturation was reached when no new topics nor information surfaced from the focus groups. One way to reach data saturation is to ask whether there are any unresolved issues from the focus group.

The researcher’s online Zoom account was used to conduct the FGD. Participants who were not speaking were muted to reduce noise while someone was speaking. Participants were invited to repeat what was said when the signal was clear again if signal interference was present. The atmosphere could be managed during the FGDs, and there were no issues with internet connectivity.

### Analysis and Data Validity

Data analysis was conducted in 2 stages: (1) inductive method and (2) triangulation with the deductive method, which was verified and validated based on the reviewed literature.

In the first stage, the research team used an inductive content analysis on each communication from the FGD, which they verified without looking at the existing literature. Field comments were added to the transcribed audio data. To identify any writing errors and validate the comments made by the participants, the transcriptions were read twice. This methodical approach ensured that every facet of the data was carefully considered and comprehended, producing thorough and trustworthy results. The transcription stage was carried out immediately after the FGD was completed. The researcher re-examined all statements from participants in both FGDs. The transcription, transcribing, and coding processes in stages 1 and 2 were carried out in the same steps. The facilitator retrieved words by attaching parallel keywords, then grouped and compared all codes. From these grouped keywords, the principal researcher then discussed them with a research team consisting of 3 participants in the FGD who acted as field observers and research assistants. The keyword data were compared with the final field notes to gain consensus in order to obtain themes that ensured trustworthiness and transparency and were not biased toward only one researcher.

The researchers used a thematic approach to analyze qualitative data in 2022 and 2024. After obtaining initial codes, terms, and themes from the FGDs, we evaluated and reviewed these themes by comparing them with the entire dataset to ensure they accurately reflected the views of health care providers. During this stage, we analyzed the themes as they evolved across time periods, specifically comparing participants’ telemedicine implementation during the peak of the COVID-19 pandemic in 2020‐2022 with the subsequent postpandemic situation in 2023‐2024. We then clearly defined and named each theme to establish its specific meaning and scope in relation to the research questions.

The information was then classified, extracted to identify significant statements, and analyzed to create meanings. After categorization, themes were developed from the data in each FGD. These themes were eventually reduced to codes and incorporated into a comprehensive analysis of the studied situation. The researchers counted the communication from participants in each phase of the FGD, grouped them by the same theme, and described them in the 2022 and 2024 FGD stages. In the end, the data are presented as study findings in the form of tables and discussion points.

In order to assure the rigor of our telemedicine FGD in 2022 and 2024, we undertook member checks to ensure credibility. In this study, we clarified remarks made by participants over the phone on the decrease in telemedicine use following the COVID-19 pandemic. Additionally, we performed peer debriefing by triangulating data from articles and national policies and addressing all themes. Additionally, we maintained confirmability by exercising researcher reflexivity and closely following the FGD guidelines, and we established dependability by guaranteeing the lack of researcher bias. Our FGD topics were cross-referenced against reputable peer-reviewed articles as part of our second strategy to ensure rigor. The triangulation stage involved analysis using a deductive approach, validating the existing themes with several previously conducted studies. This method aimed to compare existing findings with those in other studies to provide a comprehensive picture both abroad and domestically. This method was expected to provide better recommendations and a more comprehensive implementation of telenursing and telemedicine.

In the assessment of the overall readiness for telemedicine implementation, this study used triangulation by combining the results of the FGDs and theoretical foundations from journal articles synthesized through the keywords readiness, telenursing, or telemedicine. All relevant articles available as full text were read and analyzed using the telemedicine readiness approach of the 5M elements. The 5M dimensions identified in this literature review were aligned with qualitative themes extracted from the FGDs. The articles were found from the year 2005 to 2025 and from the Ministry of Health regulations in Indonesia. The articles and national regulations provided basic indicators and best practices for the 5M components; meanwhile, the FGD data offered a localized, real-world context regarding stakeholder perceptions and challenges. By cross-verifying the 2 datasets, we were able to comprehensively conclude the strengths, gaps, and readiness levels of facilities for adopting telemedicine or telenursing.

The weakness in this study’s literature review is that it was not a systematic review but only used data from articles retrieved by searching research databases with the keywords telemedicine, telenursing, benefit, barrier, and COVID-19. We conducted a convenience search of journals through ProQuest and Google Scholar for articles published from 2005 through 2025. A total of 46 journal articles were analyzed and combined with the FGD results to obtain themes. Researchers reviewed the literature using unstructured (qualitative/narrative) and incidental data, involving the application of thematic and content analysis frameworks to synthesize diverse, nontraditional, and serendipitous sources. The steps included identifying core concepts through keyword extraction, underlying arguments, and key narratives. Coding was then performed, grouping insights into themes. Synthesis was then performed, connecting, comparing, and building themes. The Quality Rating System (QRS) consists of 21 explicit items covering study design, data collection, and analysis. It helps authors write better papers, assists reviewers with evaluation, and guides readers when appraising evidence, ensuring full clarity and trust in the study. Finally, the Standards for Reporting Qualitative Research (SRQR) checklist was used to improve the transparency of all aspects of the qualitative research [19].

### Ethical Considerations

All participants provided written informed consent, and the participants were aware of the research goals, advantages, and methods. The team members maintained anonymity throughout the completion of the FGD, guaranteed confidentiality, and discussed their experiences with digital implementation. The authors adhere to ethical norms when publishing the improvement study’s data by protecting both personal and organizational privacy and data confidentiality when implementing telemedicine and telenursing. This study was approved by the Ethical Board of the Faculty of Nursing, Universitas Indonesia: KET-138/UN2.F12.D1.2.1/PPM.00.02/2022 and KET 180/UN2.F12.D.1.2.1/PPM.00.02/2024.

## Results

Attendance at focus groups has been an issue of concern, which was also identified in our research. FGDs were attended by 12 participants in 2022 and 11 participants in 2024 (Table 1). Participants included nurses, nurse practitioners, medical doctors, lecturers, and a lawyer. Verbatim responses were compiled per question and combined in participant groups in 2022 (code -22), and 2024 (code -24). After being delivered verbatim, at the end of the analysis, theme mapping was conducted for each question.

Participants’ statements in the 2022 and 2024 FGDs were then grouped, resulting in three themes: (1) health care providers have experience using telemedicine/telenursing, (2) telemedicine and telenursing have many benefits, and (3) telemedicine and telenursing have 5M factors that must be anticipated. Complete participant statements and keywords were grouped and grouped into themes, as described in Table 2.

**Table 1.** Focus group discussion participant characteristics in 2022 and 2024.

Year and participant code	Age (years)	Sex	Education	Job role
2022				
P1	33	Male	Nurse specialist	Lecturer
P2	51	Male	S1+ profession	Nurse practitioner
P3	33	Female	Master	Nurse
P4	49	Female	Medical doctor	Doctor
P5	43	Male	Master	Nurse
P6	70	Male	Professor	Lecturer
P7	51	Female	Master	Nurse practitioner
P8	29	Male	Master	NGO^a^
P9	44	Female	S1+ profession	Nurse
P10	36	Male	Master	Lawyer
P11	54	Male	Master	Nurse manager
P12	40	Male	S1+ profession	Nurse
2024				
P1	57	Male	Medical doctor	Medical doctor
P2	37	Female	S1+ profession	Nurse
P3	48	Female	Medical doctor	Medical doctor
P4	35	Male	S1+ profession	Nurse
P5	29	Female	S1+ profession	Nurse
P6	37	Male	S1+ profession	Nurse
P7	30	Female	S1+ profession	Nurse
P8	36	Female	S1+ profession	Midwife
P9	51	Female	S2+ profession	Nurse practitioner
P10	32	Female	S1+ profession	Nurse
P11	47	Female	Medical doctor	Medical doctor

a NGO: nongovernmental organization.

**Table 2.** Telenursing and telemedicine themes during and after the COVID-19 pandemic.

Themes and years	Supporting input
Health care providers have experience using telemedicine/telenursing.	
2022	Terminology telenursing and telemedicine (P6) Complement of digital transformation (P4; P6)
2024	Telenursing part of telemedicine (P1) Telemedicine and insurance (P10) As digital transformation (P1)
Telemedicine and telenursing have benefits.	
2022	Education for wound care (P2; P4) Support for education and consultation (P1; P6; P7; P9) Facilities for religious and spiritual studies (P1; P6; P9; P7) Decrease direct cost (P9; P7) Facilitating access and speeding up the consultation (P1; P6; P9; P7) Consultation and education (P6; P7; P8) Send medication to a patient in isolation (P2) Decreasing virus transmission (P1; P2; P6; P7; P8) Consultation during the pandemic (P2; P6; P7; P8) Monitoring and follow-up (P4; P11)
2024	Complement digital transformation (P1; P2; P3) Consultation and education (P2; P3; P4; P8) Facilitating access (P1; P8; P9) Decrease cost for direct treatment (P9) Referral from public health services to hospital (P4; P6) Support for education, consultation, and disaster conditions (P4; P8; P9) Education for wound care (P2:P4:P9)
Telemedicine and telenursing have 5M^a^ factors that must be anticipated.	
Method factors	
2022	Method of delivery (P1; P5; P11; P12) Payment guide (P7; P10-11) Security and privacy (P6; P10; P11; P3) Ethical conduct guide (P6; P11) Health services credentialing (P10; P5) Satisfaction and comfort (P9) Not for all, combination, education, follow-up care (P6; P10; P8) Method: need caring (P1; P12; P5: P11)
2024	Regulation (P1; P4; P9; P10) Payment national guide (P1; P4) Security and privacy (P1; P4) Kind of services Not for all services, education, follow-up care (P1; P8; P9; P11)
Man factors	
2022	Competencies (P1; P5; P9; P7) Credentialing for health provider (P10; P5) Capabilities (P1; P3; P5; P6) Training and socialization (P5; P1; P3; P6)
2024	Competencies, training, and socialization (P1; P2; P3; P4; P9) Capabilities (P1; P2; P3; P4; P9)
Money factors	
2022	Training cost (P5; P1; P3; P6) Infrastructure and investment (P8)
2024	Training cost (P1; P2; P3; P9) Infrastructure and investment (P4; P1; P2; P5; P7)
Material and machine factors	
2022	Infrastructure for integrated video calls and Zoom (P8; P10) Access (P4; P7) Network and teledevice (P8; P10; P11) Health facility credentialing (P10; P5) Integrated with EMR^b^ (P10; P3)
2024	Infrastructure: chat, AI^c^, device, application, EMR (P4; P1; P2; P5; P7) Network and teledevice (P7) Integrated with EMR (P1)

a 5M: people/man, methods, money, materials, and machines.

b EMR: electronic medical record.

c AI: artificial intelligence.

The first theme was “health care providers have experience using telemedicine/telenursing.” Participants discussed that telenursing was part of telemedicine. The existence of telemedicine and telenursing is already largely known by stakeholders. FGD participants in both 2022 and 2024 familiar with telemedicine and telenursing that help with patient care used the technology. Some implemented it in hospitals, intensive care units, and health centers, as described in Textbox 1.

Textbox 1. **Participant input supporting the “health care providers have experience using telemedicine/telenursing” theme.**

- Terminology in the health regulation; telemedicine is a clinical service provided by a health service provider through or using information technology.; telenursing as a part of telemedicine (P1-24; P6-22).
- Previously, a telenursing and telemedicine for intensive care units were built in collaboration with the Japan International Cooperation Agency. It is no longer running (P8-24).
- Telemedicine in primary health care following Badan Penyelenggara Jaminan Sosial Kesehatan assurance (P10-24)
- Telemedicine or telenursing will complement the digital transformation in Indonesia (P4-22; P6-22; P1-24).
- It was explained that, in the future, there will be development of telenursing and telemedicine (P4-24).

The second theme was “telemedicine and telenursing have many benefits.” The benefits of telemedicine and telenursing include increasing access to services. During the pandemic, it could help reduce the transmission of COVID-19. In addition, it can help with educational media, consultations, wound care, follow-up care, and spiritual care reinforcement. Participant inputs that represent this theme are shown in Textbox 2.

Textbox 2. **Participant input supporting the “telemedicine and telenursing have many benefits” theme.**

- Speed up consultations, especially during the COVID-19 pandemic (P1-22; P2-22; P6-22; P7-22; P8-22)
- Decreasing virus transmission during the COVID-19 pandemic; send drugs (P6-22; P7-22; P8-22; P2-22)
- Consultation, education (P6-22; P7-22; P8-22; P2-22; P2-24; P3-24; P4-24; P8-24)
- Telenursing will bring patients closer to nurses, facilitating access. The service is without walls (P1-22; P6-22; P9-22; P7-22; P1-24; P8-24; P9-24).
- Telenursing will decrease costs for direct treatment (P9-22; P7-22; P9-24).
- Make referrals easier from public health services to hospital (P4-24; P6-24)
- Telenursing can be used for long-distance wound care consultations. Consultations from the disaster location are also useful during disasters (P2-22; P7-22; P8-24).
- In addition to education, this application also facilities religious and spiritual studies (P1-22; P6-22; P9-22; P7-22).
- This is to support education for patients with wounds care (P2-24; P4-24; P9-24).
- Telenursing is very useful for monitoring and following up patients after being treated at the hospital (P4-22; P11-22).
- During COVID-19 virtual consultations, nurses first access their patients, then after being assessed, they are given education related to their nursing; they then consult a medical doctor. The doctor makes a diagnosis, then they are given a new prescription order to the pharmacy, and the medicine will be sent via application parties (P8-22).

The third theme was “telemedicine and telenursing have 5M factors that must be anticipated.” The weaknesses of telemedicine and telenursing were identified by participants. The main problem was the incomplete national regulations on service rules through technology, including payment. Human resource competence, connection, and infrastructure readiness were still an issue. Several participants said that it, especially telenursing, cannot be implemented entirely based on technology. Nursing care must still have contact with the patient as a manifestation of caring from nurses to patients.

The expectations of participants toward the implementation of telemedicine and telenursing were synthesized using the 5M approach, namely method, man, money, material, and machine. In the method category, management of telemedicine and telenursing is needed. This includes selecting services that can use tele/digital services, including education, monitoring, follow-up care, providing motivation, and spiritual aspects. Some services that require direct contact and diagnosis enforcement still require a dual-method model, namely direct and technology-based services. Moreover, telemedicine and telenursing services must also prioritize patient and family satisfaction and comfort. There is a need for health facility credentials to permit the delivery of a telemedicine or telenursing program. Human resources must also have the authority for technology-based services and to receive training to improve digital literacy. Money should be set aside specifically for infrastructure upgrades, for training to use the equipment, and to increase digital literacy. Participant input that represents this theme is described in Textbox 3.

Textbox 3. **Participant input supporting the “telemedicine and telenursing have 5M (man, methods, money, materials, and machines) factors that must be anticipated” theme.**

- I hope this telenursing will make it easier for patients to access it (P4-22; P7-22).
- The competencies that must be possessed by this telenursing; are specialist nurses allowed to do tele-nursing nursing or nurses with a minimum of S1 (Bachelor nursing) or D3 (diploma)? (P4-24; P9-22)
- Need credentials for human resources and health facilities that organize telemedicine (P10-22; P5-22)
- It is necessary to improve the human resource capabilities of both doctors and nurses; perhaps it needs to be socialized if there is an application…training cost (P5-22; P1-22; P3-22; P6-22; P1-24; P2-24; P3-24; P9-24).
- For telenursing method of delivery, not all care can be done by tele. Nurses need direct touch; caring becomes an issue when only via tele (P1-22; P12-22; P5-22, P11-22).
- What platform will be used in telemedicine or telenursing; must prepare it? Cost for infrastructure and investment (P4-24; P1-24; P2-24; P5-34; P7-24; P8-22)
- Network problems are not all good; not all areas have teledevices (P8-22; P10-22; P12-22; P11-22; P7-24).
- To data security, it must be integrated with electronic medical record (EMR; P10-22; P3-22; P1-24).
- Regulation must clear; payment must be managed by national regulation (P7-22; P11-22; P1-24; P4-24).
- We need to push for national guidelines for the implementation of telemedicine and telenursing (P10-22).
- Need professional ethical guidelines in using telenursing-based services (P6-22; P11-22)
- It’s not a clear condition. I've never seen the exact regulation, like payment regulations. But, as far as I know, already a regulation. I just haven't read it (P9-24; P10-24).
- Regulations regarding telenursing or telemedicine services and payments are not yet clear (P1-24; P4-24).
- Even though virtual is used, it must guarantee patient satisfaction and comfort (P9-22).
- Suggestions for telenursing should choose a type of service that can use digital technology. Monitoring, follow-up care, or education can be done with telenursing. This activity is still combined with direct meetings between nurses and patients (P6-22; P10-22; P8-22; P1-24; P8-24; P9-24; P11-24).
- Not all treatments can be done with telenursing such as wound care. Consultation and education can be done with telenursing or telemedicine (P1-22; P5-22; P11-22; P12-22).
- But it’s necessary to follow up on the patient’s condition, such as using video calls, Zoom to be able to carry out a more in-depth assessment (P8-22; P12-22).

Complete participant statements and keywords were grouped into themes, as described in Table 2.

Table 2 describes the keywords used in 2022 and 2024 that were compiled into themes derived from FGDs related to telenursing and telemedicine. There are differences in emphasis: in 2022, heavy focus was placed on the benefits of telenursing and telemedicine reducing the transmission of COVID-19, whereas in 2024, the benefits of telemedicine expanded to include access, education, and consultations. The 5M factors were described almost identically by the participants in 2022 and 2024. In this study, researchers also calculated keywords used to identify trends or provide support for themes and validated using incidental studies found in the literature (Table 3).

**Table 3.** Validation between the focus group discussion (FGD) themes and the literature review.

Themes and supporting FGD participant statements		Supporting participant statements in 2022 (n=23), n (%)		Supporting participant statements in 2024 (n=23), n (%)		Supporting literature
Telemedicine and telenursing have many benefits.						Song et al [2 ], Almahroos et al [20 ], Tort-Nasarre et al [1 ], Smith et al [3 ], Nejadshafiee et al [4 ], Hariyati et al [8 ], Moriyama et al [9 ], Ariyanto and Rosa [11 ], Hjelm [13 ], Li et al [12 ], Haimi [14 ], Costantini et al [21 ], Main et al [22 ], Zhang et al [23]
Complement digital transformation		3 (13)		3 (13)		
Speeding up and facilitating access to consultations		5 (22)		4 (17)		
Decreasing virus transmission, send drugs, consultation, and education		4 (17)		1 (4)		
Bringing patients closer to nurses, facilitating access to hospital referrals		2 (9)		4 (17)		
Decreasing indirect and direct costs		2 (9)		1 (4)		
Wound care and disaster consultation		2 (9)		3 (13)		
Facilities for religious studies		4 (17)		0 (0)		
5M^a^ factors						Hjelm [13], Hariyati et al [6], Kennedy and Yaldren [24], Boro and Hariyati [7], Reed et al [25], Hwei and Octavius [15], Alexander et al [26], Moulaei et al [18], Hariyati et al [8], Wang and Cao [17], Liu et al [27]; Lee and Tak [28], Haimi [14], Tort-Nasarre et al [1], Zhang et al [23]; Mattisson et al [29], Sri Hariyati et al [30]; Vidal-Silva et al [31], Li et al [12], Hariyati et al [16]
Man						
Competencies: training, socialization		4 out of 17 (13%)		3 (13)		
Credentialing for health care providers		2 (9)		0 (0)		
Method						
Regulation and national guidance, payment guide		4 (17)		5 (22)		
Security and privacy		4 (17)		2 (9)		
Ethical guide		2 (9)		0 (0)		
Health care services credential		2 (9)		0 (0)		
Not all can be done/combination with telenursing		4 (17)		4 (17)		
Money						
Training cost		4 (17)		4 (17)		
Infrastructure investment		1 (4)		4 (17)		
Material and machine						
Network problems, devices, application		3 (13)		3 (13)		
Data security, data confidentiality		3 (13)		3 (13)		

a 5M: people/man, methods, money, materials, and machines.

**Figure 1.**
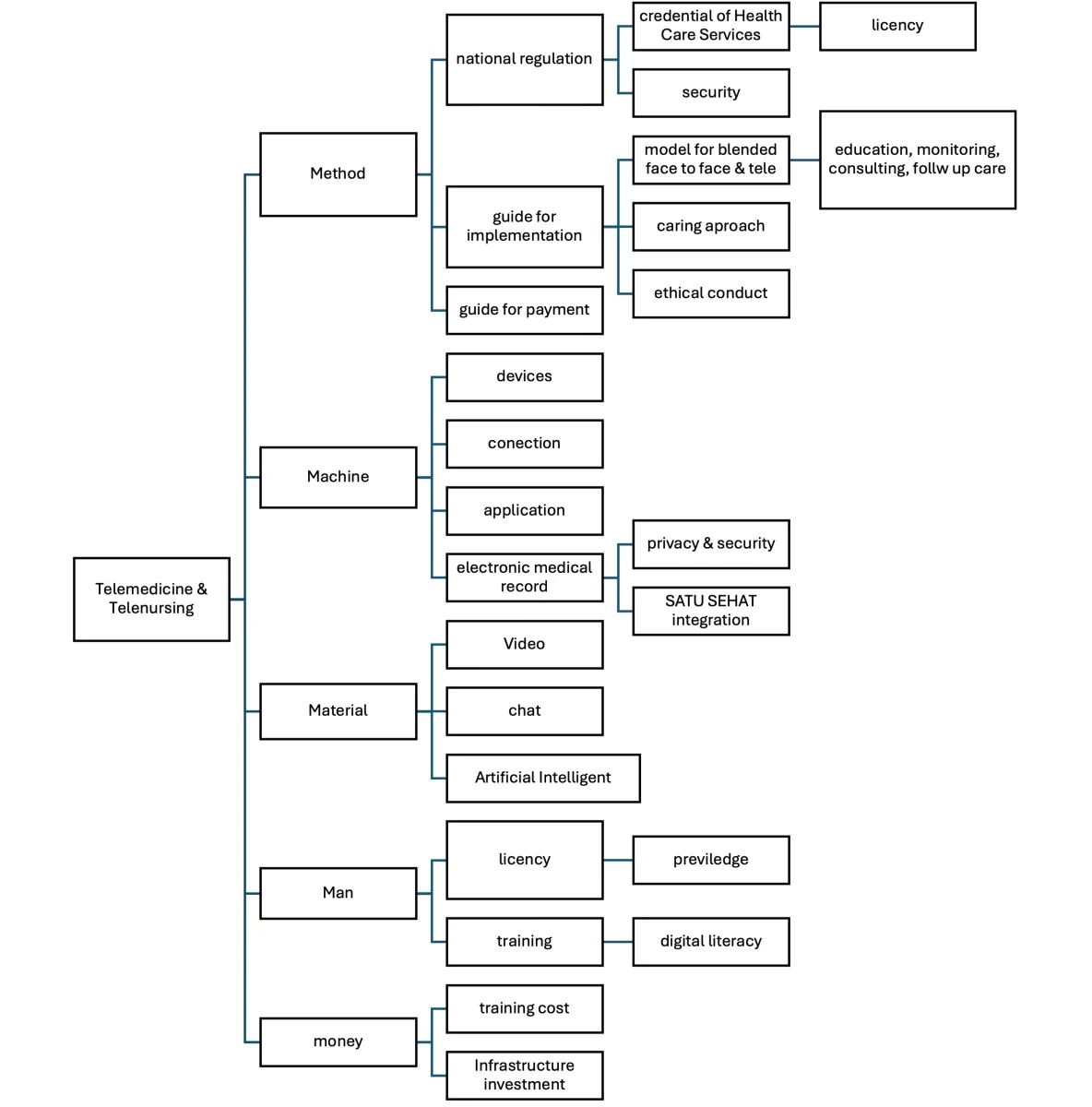
Recommendation for better implementation of telemedicine and telenursing.

Table 3 gives details of the analysis comparing the journal articles and the description of subthemes and themes. We also provide the percentage of participants who conveyed similar meanings of the sentences. All topics are represented in the theme table, which was built by combining the synthesis of the journal articles and participant statements. After understanding the advantages and disadvantages of telenursing, recommendations were made for the implementation of telemedicine. The recommendations were developed using the 5M approach (Figure 1).

As shown in Figure 1, the method includes telemedicine and telenursing management, as well as the selection of tele/digital services that can be used, such as education, monitoring, follow-up care, motivation, and spiritual aspects. This represents a dual direct and indirect model using a technology-based service that still maintains direct contact for certain services such as diagnostic confirmation. Credentials for health care facilities authorized to conduct telemedicine or telenursing must be obtained from the health facility side. Patient service documentation must be arranged so that it securely uses electronic medical records (EMRs), which must be integrated with national data from SATU SEHAT Indonesia in the future. The availability of facilities including telemedicine equipment and internet connections must continue to be developed so that they can reach all Indonesian islands. Additionally, human resources must be in charge of technology-based services and undergo training to increase their digital literacy level.

## Discussion

### Principal Findings

This research explored the experiences of health care stakeholders using telemedicine and telenursing. We identified the following three themes: (1) health care providers have experience using telemedicine/telenursing, (2) telemedicine and telenursing have many benefits, and (3) telemedicine and telenursing have 5M factors that must be anticipated. The research results indicate that telemedicine has been implemented in Indonesia, and its use increased during the COVID-19 pandemic. Although some participants mentioned that the hospitals where they work had not yet implemented telemedicine, all participants were aware of the implementation of telemedicine. Telenursing is also one type of telemedicine that is widely used by nurses for follow-up care, monitoring, wound care consultations, and providing motivation.

The results of the research analysis describe many benefits of telemedicine, especially considering Indonesia’s archipelagic condition. Telemedicine itself is a health care service for remote examinations, diagnosis determination, and therapy administration remotely, aided by technology such as phones, video conferences, and other methods [2,7]. Differences in emphasis were observed: in 2022, the advantages of telenursing and telemedicine centered primarily on decreasing the transmission of COVID-19, whereas by 2024, the benefits of telemedicine had expanded to encompass access, education, and consultations. With the help of technology, access to services will be improved, and patients will be brought closer to health care services without having to go to hospitals or community health centers. This study supports the statement that telemedicine helps rural areas access health care services, in line with previous research [14,15,20,32].

The ability of technology to reach various rural areas means that telemedicine and telenursing can reduce both direct and indirect costs. This has also been conveyed by previous research, which states that, if consultations are conducted online, travel costs to the hospital can be saved; additionally, it will save indirect costs such as those caused by time inefficiency [14,21-23]. Another previous study also stated telestroke was a highly cost-effective strategy, which may enhance stroke care and improve favorable outcomes in patients in rural China [33].

In addition to the benefits, telemedicine also faces challenges in its implementation. The participants provided many recommendations to anticipate the 5M barriers to implementation. The obstacle of implementation failure must be addressed by creating national regulations related to telemedicine and telenursing [14]. In 2023, Indonesia started requiring the use of EMRs, which aims for data integration and interoperability toward one data called SATU SEHAT (ONE HEALTH) Indonesia [34]. Telemedicine and telenursing must also begin to be integrated into SATU SEHAT and become part of the digital transformation in the health sector. Policies ensuring the security and confidentiality of EMR data must be enforced. This is in accordance with the synthesis of studies stating that medical records must document telemedicine-based service results and that data security must be maintained [34,35].

Another recommendation regarding policy is that credentialing requirements must be established for telemedicine providers. Only institutions with permits are allowed to conduct telemedicine or telenursing. In Indonesia, a regulatory sandbox was required, and all electronic-based health care service providers should be registered and assessed for feasibility before implementing telemedicine [35]. So this policy can be implemented, the government must be encouraged to apply it according to the regulations [14,17,35]. In addition to the organizing bodies that must have permits, the human resources providing care must be credentialed and licensed to practice telemedicine or telenursing. Nurses and doctors who use technology must receive training to improve digital literacy [8,16,36]. This digital literacy is essential for optimal telenursing performance.

Barriers that need to be anticipated are also related to telemedicine facilities. Text, video, telephone, and AI-based consultation media must be developed thoroughly [21,37,38]. The use of video facilitates the interaction between nurses and patients. The use of video also allows health care workers to monitor patients’ conditions, and decisions will be faster with the help of AI [5,39,40]. Unfortunately, sophisticated technology often becomes a barrier for patients who lack digital literacy. Many older adult patients and vulnerable individuals do not have the tools for telemedicine, which can hinder the development of telemedicine and telenursing [10,27,41]. In addition to the tools, network connections are not evenly distributed in every region. Therefore, previous research recommends that the government strengthen the network connections to ensure the successful implementation of telemedicine [12-14,42].

Not all care can be perfectly delivered through telemedicine. Some participants stated that they had to choose the type of service that could be provided through telenursing but still used a dual approach, namely face-to-face consultation followed by telenursing. Nurses, whose core responsibility is caring, must continue to prioritize care that consistently enhances satisfaction and comfort [8,29,43].

Patient and family preferences also significantly influence the success of telenursing. The paradox of telenursing and telemedicine, where there is no direct contact with health care personnel, means there is no physical touch, and the fear of misassessment often leads to hesitation in choosing the telemedicine method [18,26]. Therefore, it is recommended to maintain deep interactions [29] and enhance the sophistication of tools that can monitor patient conditions such as vital signs, for example, with heart rate monitors that automatically connect to servers [33,44]. Digital literacy education for the community must also be continuously strengthened so that the community increasingly understands the benefits of telenursing or telemedicine [24,28]. Improving digital literacy for the community can help them choose telemedicine and telenursing as an alternative support option for health care services [25]. Enhancing social impact is another recommendation for telemedicine success, as it can lessen apprehension about using new technology, particularly if the patient and health care practitioner have had favorable experiences with telemedicine [30]. The role of manager in encouraging the use of telemedicine is also very important, such as how managers controlled infections during COVID-19 by modifying services using technology. They must also be innovative and be role models in implementing telemedicine.

### Limitations

This study used a mixed approach to analyze the qualitative descriptive results from the FGDs and journal articles. The weakness of this study is that the journal search used a convenience approach and was not part of a systematic review. The FGD participants in 2022 and 2024 were not the same people, so follow-up on the development of telenursing and telemedicine was not possible. Although it has weaknesses, the results of this study can be used as initial findings to improve the implementation of telemedicine in Indonesia and other countries that have facilities, resources, and management that are almost the same as Indonesia. In future research, a systematic review and an in-depth interview method should be conducted.

### Conclusion

These findings highlight the advantages and disadvantages of telemedicine and telenursing. We identified the following three themes from the study results: (1) health care providers have experience using telemedicine/telenursing, (2) telemedicine and telenursing have many benefits, and (3) telemedicine and telenursing have 5M factors that must be anticipated. The study’s findings suggest that 5M factors should be prepared to implement telemedicine. These include procedures like payment plans, human resource credentials, and licensing rules for organizers. Enhancing digital literacy for the community and health professionals is also recommended, as well as creating a budget that focuses on infrastructure improvements and training for technology use and digital literacy. For telemedicine to function well, upgrading facilities and adding internet access are required. A service type must be selected to generate a service recommendation, which may involve a combination of telemedicine and in-person care, such as an initial in-person visit to a health care facility followed by monitoring via telenursing. Telenursing and telemedicine can help with activity monitoring, follow-up treatment, patient and family education and consultation, and incentive building. Enhancing patient satisfaction and care must be a priority when providing nursing care via telenursing. There are implications for governance; operational rules for telemedicine and telenursing must be developed, and health care providers must increase their digital literacy.

## Abbreviations

5M: man, money, method, material, and machine
EMR: electronic medical record
FGD: focus group discussion
QRS: Quality Rating System
SRQR: Standards for Reporting Qualitative Research
